# The Impact of the Beirut Explosion on the Mental Health of Lebanese Healthcare Providers: A Scoping Review

**DOI:** 10.7759/cureus.74240

**Published:** 2024-11-22

**Authors:** Mariana Helou, Danielle Abou Khater, Fadi El Ters, Kaissar Yammine

**Affiliations:** 1 Department of Emergency Medicine, Lebanese American University School of Medicine, Beirut, LBN; 2 Department of Orthopedic Surgery, Lebanese American University Medical Center, Beirut, LBN

**Keywords:** beirut explosion, healthcare workers, mental health, psychological distress, public mental health

## Abstract

Lebanon has faced a series of crises, starting with the economic collapse in 2019, followed by the COVID-19 pandemic, and the Beirut blast on August 4, 2020. These events have left the population vulnerable to psychological distress. Our study aims to assess the psychological impact of the Beirut explosion on Lebanese healthcare providers. We conducted an electronic database search, resulting in the inclusion of 21 relevant manuscripts. Various psychological issues were reported among healthcare workers (HCWs), with burnout prevalence rates of 37.2% for disengagement and 51.5% for exhaustion. Additionally, feelings of heaviness, fear, exhaustion, and anxiety were common. Approximately 60% of HCWs experienced moderate to severe stress, and half reported moderate to severe anxiety and depression. Furthermore, 44% were at high risk of developing post-traumatic stress symptoms. Lebanese HCWs have faced significant psychological trauma in recent years, underscoring the need for mental health awareness campaigns and targeted training for HCWs.

## Introduction and background

Lebanon, a small middle-income country in the Middle East, has faced a series of consecutive crises within a one-year period, including prolonged political instability, massive riots, and the unprecedented devaluation of its currency in October 2019 [1,2]. The financial crisis that began in late 2019 escalated throughout 2020 and continues to have a profound impact today [3]. This crisis has significantly disrupted all sectors, particularly the healthcare sector [3].

The political instability in Lebanon has been compounded by inadequate regulations and concerns over the safety of residents, resulting in a major deterioration in the psychological well-being of the population [4]. Healthcare workers (HCWs) have been similarly affected by the financial crisis, with rising inflation, resource scarcity, insecurity, and increased workloads contributing to mental health challenges, ranging from anxiety to burnout [5,6]. The income of physicians has also been significantly impacted, further exacerbating the stress they face [7].

On March 11, 2020, the WHO declared COVID-19 a global pandemic [8]. The virus reached Lebanon in early 2020, and within three months, the increasing number of cases overwhelmed the healthcare system [1]. At that time, Lebanon was already grappling with the aforementioned economic crisis and lacked sufficient resources to effectively manage the pandemic [9]. The mental health of young people was particularly affected, with an increase in cases of depression, anxiety, and insomnia [8]. The isolation measures implemented to control the spread of the virus also had significant psychological effects on both patients and HCWs [10]. Several studies have highlighted the mental health toll of these measures, showing elevated levels of anxiety, depression, and fear among HCWs [10]. A study conducted in a tertiary care center in Lebanon revealed that half of the participants, including physicians and nurses, reported a high risk of acute distress [9].

On August 4, 2020, Lebanon was struck by a devastating explosion at the Beirut port, caused by the detonation of approximately 2,750 tons of ammonium nitrate. This blast claimed the lives of 235 people, injured thousands, and displaced 300,000, leaving much of the capital city in ruins. The explosion has made the Lebanese population highly vulnerable to psychological trauma. Several studies have discussed the psychological impact of the blast on the population, with post-traumatic stress disorder (PTSD) [11-15], depression [13-15], anxiety [13,15], and even eating disorders [11] being reported. A study in 2022, involving 703 participants, used the SCOFF questionnaire to screen for eating disorders and the IES-R questionnaire for PTSD. It found that 42% of participants had a positive PTSD score, with a significant correlation between exposure to the explosion and PTSD symptoms. Furthermore, 32% of participants screened positive for eating disorders, with significant associations found between eating disorders and factors such as relationship status, education level, mental illness history, and degree of exposure to the explosion [11]. A correlation was also noted between PTSD and eating disorder scores. Another study, conducted three months after the blast on 996 Lebanese residents, revealed a link between negative religious coping and higher levels of PTSD symptoms, particularly among females, residents of Beirut, those who had been injured, or those who knew someone injured in the explosion [12].

This study aims to assess the psychological disturbances experienced by Lebanese healthcare providers in response to the Beirut blast.

## Review

Methods

Search Strategy

The electronic databases of the Cochrane Library, Medline, Embase, and Google Scholar were searched using the following keywords, combined with Boolean terms: [Beirut AND (explosion OR blast) AND (nurse OR physician OR resident OR fellow OR postgraduate OR undergraduate)]. Articles published between January 2020 and May 2022 were considered. Additionally, governmental and nongovernmental reports were reviewed. No language limitations were applied. A total of 21 manuscripts were included in this report. Institutional Ethics Committee approval was not sought, as the study design falls under the review category.

Study Criteria

All study designs, except case reports, were accepted for inclusion.

Results

A total of 21 manuscripts were initially identified for the study. After reviewing the titles and abstracts, 14 manuscripts were deemed suitable for inclusion in our review.

Burnout

A study conducted on postgraduate medical trainees (PGMTs) in a medical facility in Lebanon, which included 20 residency programs, found that the prevalence rates of burnout were 37.2% for disengagement and 51.5% for exhaustion. These rates varied significantly across different years of training, with the highest levels observed in postgraduate year (PGY) 3 and the lowest in PGY 7. No gender differences were found in burnout rates. Additionally, 65% of residents expressed hesitation in reporting their burnout, with the primary contributing factors being working hours, salary, and work conditions [16]. Another study in Lebanon, which surveyed 188 PGMTs, reported a prevalence of 68.6% for personal burnout, 63.3% for work-related burnout, and 35.1% for patient-related burnout [17]. A significant gender difference was observed in personal burnout, with females exhibiting higher rates.

Anxiety and Stress

The prevalence rate of moderate to severe stress is 58.1% among HCWs and 69% among medical students. The prevalence of moderate to severe anxiety is 48.7% and 46.8%, respectively, with higher rates observed in women and those with low monthly incomes [18]. Following the Beirut Blast on August 4, 2020, the Lebanese population, particularly healthcare providers who were on the frontline, experienced significant psychological distress. Healthcare providers reported feelings of heaviness, anxiety, fear, exhaustion, and trauma [19,20]. They also described sensations of worry, tears, sadness, and sleepless nights [21]. Nurses, in particular, expressed feelings of helplessness, anger, fear of death, and persistent overthinking, often coupled with a depressive mood [22]. Some nurses relived the August 4 incident through flashbacks and panic attacks [22].

Depression

A cross-sectional study involving 374 medical students and HCWs revealed that approximately 70% of medical students and 60% of HCWs experienced moderate to severe stress [18]. Additionally, around 50% of the participants reported moderate to severe anxiety and depression, with these symptoms being more prevalent among women.

Post-Traumatic Stress Symptoms

A study conducted nine to 15 days after the Beirut Blast on 570 HCWs found that the prevalence of acute stress disorder was 38.34% [23], with nurses and females being at higher risk. Another study, conducted in December 2020 on 519 HCWs at a private hospital in Beirut, revealed that 44% were at high risk of developing post-traumatic stress symptoms [24]. Surgeons, emergency physicians, anesthesiologists, and radiologists were identified as being at higher risk.

Discussion

The COVID-19 crisis, exacerbated by Lebanon’s ongoing financial turmoil, overwhelmed both the government and the healthcare system. On August 4, 2020, a massive explosion at the port of Beirut further devastated the country. Forty percent of Beirut was demolished, including numerous homes, leaving 300,000 people homeless. Over 6,500 individuals were injured, and 200 lost their lives [25-27]. This overwhelming destruction strained hospitals, and the explosion of medical supplies and personal protective equipment further worsened the healthcare crisis [27]. Both material losses and physical damage were extensive [27]. Psychiatric disorders typically worsen following disasters [28], and despite the traumatic consequences of the explosion, many individuals were required to return to work the following day. This posed a significant challenge for HCWs, who were expected to suppress their emotions and traumas while contributing to the community's recovery [29]. Data on mental health disorders in Lebanon post-blast remain limited, though one study noted an increase in psychological symptoms among patients attending private hospital clinics [30]. Given that the explosion was deemed an act of terrorism, widespread feelings of anger and suffering emerged [31].

Burnout rates vary across countries. For instance, in Pakistan, 49.74% of PGMTs reported personal burnout [32], while in India, the rate was 51.8% [33]. In the United States, one study reported a burnout rate of 46.3%, while another found it to be 35.8% [34,35]. In Brazil, 48.6% of PGMTs reported burnout [36], and in France, the rate was 37.4% [37]. These differences are influenced by factors such as variations in measurement tools, PGMT specialties, and the local sociopolitical environment.

Burnout levels vary across medical specialties, with those involving higher exposure to emergencies and greater daily stress tending to report higher burnout levels. Additionally, several studies highlight higher burnout rates among female trainees. This may be attributed to the additional burdens faced by women, particularly those who are married or have children, as they often bear increased responsibilities both at home and in their professional roles.

Trainees often do not report burnout, possibly due to a belief that reporting will not lead to meaningful change. Studies consistently show that stress symptoms are more prevalent after man-made disasters than natural ones. The prevalence rate following the Beirut Blast was similar to that observed after the 9/11 World Trade Center attack, at 42% [38]. In contrast, the rate of PTSD among HCWs during pandemics ranged from 16.7% to 40% [39-41].

Due to Lebanon’s inadequate mental health services and limited governmental funding, the Ministry of Public Health launched a mental health program providing psychological support and counseling for individuals impacted by the Beirut explosion [42].

Mental Health Policies

As highlighted in our results, Lebanon has faced three consecutive disasters. The first was the country’s economic and political collapse, leading to inflation, rising unemployment, and poverty, with limited access to basic necessities. This crisis had a profound psychological impact on the Lebanese population. Amid the financial collapse, the COVID-19 pandemic emerged, exacerbating the situation. Due to the ongoing economic crisis, the burden of COVID-19 was heavier in Lebanon compared to other countries. In addition to the anxiety and fear surrounding the new virus, the population also faced concerns about their inability to access essential healthcare and provide for their families during lockdowns. The psychological well-being of the Lebanese people further deteriorated after the catastrophic explosion in Beirut.

In Lebanon, there are no comprehensive mental health support policies, and the country faces a shortage of psychiatrists and psychologists, with 90% of mental health issues being inadequately treated [7]. Studies have shown that 17% of Lebanon’s population is affected by psychiatric conditions, such as anxiety, stress, depression, and other mental health disorders [43,44].

Overall, Lebanon’s financial collapse, compounded by the COVID-19 pandemic, significantly increased stress and anxiety levels among its population [45]. After the lockdowns, individuals reported a deterioration in their mental health [46]. Following the Beirut blast, most individuals experienced daily worry and anxiety, with a persistent sense of threat [47]. Many of these individuals recognized their mental health struggles and sought mental health services [48]. Furthermore, a study of young people in Lebanon revealed that 11.5% had experienced suicidal ideation [49]. Notably, suicide rates in Lebanon were the highest in 2020 and 2021 [50-52].

Mental health services in Lebanon are not widely accessible, and when available, they are primarily concentrated in the capital, Beirut. Community-based services are scarce, and HCWs receive little training to manage mental health cases. Only 5% of the country's general health budget is allocated to mental health [1], with most funds directed towards chronic patients in private hospitals. A 2015 WHO report highlighted these challenges, revealing that there are approximately 15 mental health professionals per 100,000 people, whereas the recommended ratio is at least 4.45 skilled workers per 1,000 people [53]. Additionally, there is a lack of awareness among the Lebanese population regarding psychological issues, the importance of mental well-being, and when to seek help. Financial constraints and limited insurance coverage for mental health care lead many individuals to prioritize physical health issues over their mental health.

International support plays a crucial role in advancing mental health initiatives in Lebanon. Recently, several projects have been launched, including the National Mental Health Program (NMHP), which provides psychological support to individuals affected by the Beirut Blast [54]. Other initiatives have also been initiated, some still in their early stages, and others targeting specific groups such as Syrian refugees. However, it is evident that further support and coordination are necessary, both at the national and international levels, to address the ongoing mental health challenges in Lebanon effectively.

## Conclusions

Lebanon has faced numerous challenges over the past decade. In a country grappling with crisis, mental health support is crucial but has not received the attention it requires. There is an urgent need for awareness campaigns about mental health, as well as training sessions for HCWs to better address these issues. Additionally, international funding and projects are essential to support mental health initiatives and ensure the well-being of the population.
